# Tislelizumab-induced immune-mediated hepatotoxicity combined with two episodes of hepatitis B virus reactivation: a case report

**DOI:** 10.3389/fphar.2026.1951692

**Published:** 2026-10-13

**Authors:** Dongxuan Li, Guiqin Peng

**Affiliations:** 1 Department of Pharmacy, The Third Affiliated Hospital of Chongqing Medical University, Chongqing, China; 2 Department of Pharmacy, Chongqing University Cancer Hospital, Chongqing, China

**Keywords:** tislelizumab, immune-mediated hepatotoxicity, hepatitis B virus reactivation, glucocorticoid, case report

## Abstract

Immune-mediated hepatotoxicity (IMH) is a typical immune-related adverse event associated with the use of immune checkpoint inhibitors (ICIs), while hepatitis B virus reactivation (HBVr) is an infectious complication that may also occur during ICI therapy. However, these two conditions are usually independent and rarely co-occur in the same individual. Herein, we describe a patient with lung squamous cell carcinoma who developed HBVr and IMH simultaneously after receiving tislelizumab, and the co-existence of two episodes of HBVr brings challenges to the diagnosis and treatment of IMH. In the diagnosis of IMH, the co-existence of HBVr masked the underlying IMH, resulting in a delay in IMH treatment. In the treatment of IMH, the re-occurrence of HBVr led to a dilemma of continuing to use glucocorticoids (GCs) or stopping them. In such a clinical scenario, the multidisciplinary approach, close monitoring of liver function and HBV-DNA, and a carefully balanced treatment strategy combining GCs with intensified antiviral therapy are essential to realize the optimal management for IMH. In conclusion, medical staff should be alert to the coexistence of HBVr and IMH in patients receiving ICIs, so as to avoid missed diagnosis and treatment delay.

## 1 Introduction

Tislelizumab is a monoclonal IgG4 antibody against human programmed death receptor −1, which is widely used in the treatment of various malignant tumors at present, including non-small cell lung cancer (Lee and Keam, 2020). Although immune checkpoint inhibitors (ICIs), represented by tislelizumab, have become an effective cancer treatment, they can also lead to a breach in self-tolerance of the patient, resulting in immune-related adverse events (irAEs) (Singh et al., 2023). The liver is one of the most frequently affected organs in irAEs, and it is reported that about 14% of patients will suffer from immune-mediated hepatotoxicity (IMH) during ICI treatment (Jiang et al., 2024). Hepatitis B virus (HBV) is a common cause of liver injury in the clinic and also a differential diagnosis that needs to be excluded before making an IMH diagnosis (Peeraphatdit et al., 2020). In clinical practice, it is not uncommon for hepatitis B surface antigen (HBsAg)-positive cancer patients receiving ICIs to experience HBV reactivation (HBVr). A retrospective cohort study reported that about 5.3% of HBsAg-positive cancer patients developed HBVr after exposure to ICIs and showed transaminase elevation (Zhang et al., 2019). However, IMH and HBVr are usually independent events that rarely occur in the same individual at the same time. Herein, we describe a patient with lung squamous cell carcinoma who developed HBVr and IMH simultaneously after receiving tislelizumab. In this case, the two episodes of HBVr greatly complicated the diagnosis and treatment of IMH, which suggested that medical staff should be alert to the coexistence of HBVr and IMH in patients receiving ICIs to avoid missed diagnosis and treatment delay.

## 2 Case description

A 61-year-old man without a history of alcohol use was diagnosed with lung squamous cell carcinoma of the right upper lobe (cT4N3M0, stage IIIC) in April 2026. He has no mutation in the EGFR/ALK/ROS1 genes, and the PD-L1 expression level is about 1%. He underwent HBV serologic testing on 25 March 2026, which revealed positivity for hepatitis B surface antigen (> 250.00 IU/mL), hepatitis B e antibody (> 3.00 IU/mL), and hepatitis B core antibody (> 10.00 IU/mL), and entecavir was initiated for antiviral therapy. On 22 April 2026, the patient received the first cycle of anti-tumor therapy, consisting of albumin-bound paclitaxel (400 mg), carboplatin (450 mg), and tislelizumab (200 mg). Before starting anti-tumor therapy, the laboratory tests showed that the liver function index of this patient was basically normal: alanine aminotransferase (ALT) 40 U/L, aspartate aminotransferase (AST) 47 U/L, total bilirubin (TBil) 6.67 μmol/L, gamma-glutamyltransferase (GGT) 32 U/L, alkaline phosphatase (ALP) 72 U/L, HBV-DNA was below the detection limit of 30 IU/mL, and the hepatobiliary sonography was also normal without liver fibrosis or cirrhosis.

On 13 May 2026, the patient plans to receive the second cycle of anti-tumor therapy. However, in the pre-treatment examination, the laboratory tests showed that the liver function index of this patient was significantly abnormal (ALT 578 U/L, AST 589 U/L, TBil 13.50 μmol/L, GGT 67 U/L, ALP 83 U/L). In addition, the HBV-DNA detection result is 1320 IU/mL, which indicates that HBV was reactivated. Due to liver injury and HBVr, the anti-tumor therapy was postponed. Meanwhile, considering that entecavir had been unable to control HBV replication and HBV had the risk of further activation, tenofovir disoproxil fumarate was used instead of entecavir to strengthen anti-HBV therapy. Seven days later (20 May 2026), the laboratory tests showed the HBV-DNA quantification returned to the lower detection limit (< 30 IU/mL) again, and the liver function index of this patient has also declined significantly (ALT 240 U/L, AST 258 U/L, TBil 11.55 μmol/L, GGT 131 U/L, ALP 126 U/L), suggesting HBVr is the cause of liver injury. Therefore, the patient continued to take tenofovir disoproxil fumarate, waiting for the recovery of liver function to restart anti-tumor treatment.

However, the patient’s liver function index did not decrease as expected in the laboratory tests conducted on 24 May 2026 (ALT 237 U/L, AST 284 U/L, TBil 12.62 μmol/L, GGT 157 U/L, ALP 138 U/L) and 27 May 2026 (ALT 229 U/L, AST 277 U/L, TBil 16.89 μmol/L, GGT 163 U/L, ALP 138 U/L). Instead, they remained at a relatively high abnormal level, which made us suspect that tislelizumab is another important cause of liver injury in this patient. Instantly, the patient started an intravenous drip of 30 mg methylprednisolone twice a day to treat ICI-related hepatotoxicity. Four days later (1 June 2026), the laboratory tests showed that the liver function index of this patient declined significantly (ALT 166 U/L, AST 77 U/L, TBil 8.13 μmol/L, GGT 149 U/L, ALP 145 U/L), but the HBV-DNA quantification (178 IU/mL) was detectable again. The above results indicated that glucocorticoid (GC) treatment was effective, but it also served as a trigger for HBVr. To determine the subsequent treatment plan, a joint consultation involving gastroenterologists, oncologists, and clinical pharmacists was carried out. After the discussion, we reached a consensus that continuing GC therapy is beneficial to restore liver function and restart anti-tumor therapy, while the risk of HBV activation is relatively controllable under the setting of gradually decreasing GC and strengthening antiviral therapy. Therefore, this patient continued with GC therapy and started antiviral treatment using tenofovir disoproxil fumarate in combination with entecavir.

On 5 June 2026, the patient had liver function and HBV-DNA quantification rechecked. The laboratory tests showed that the patient’s liver function continued to improve (ALT: 105 U/L, AST: 57 U/L, TBil: 6.48 μmol/L, GGT: 126 U/L, ALP: 132 U/L), but the HBV-DNA quantification (439 IU/mL) increased slightly. This mild increase in HBV-DNA did not prevent the implementation of GC therapy, and the patient continued a GC dose-ladder reduction regimen in the following weeks (Table 1). On 3 July 2026, the patient finished GC therapy. The laboratory tests on 6 July 2026 showed that the patient’s liver function had returned to near-normal levels (ALT: 53 U/L, AST: 60 U/L, TBil: 7.29 μmol/L, GGT: 38 U/L, ALP: 90 U/L) and the HBV-DNA quantification also dropped below the detection limit (< 30 IU/mL), meeting the criteria for resuming anti-tumor therapy. On 8 July 2026, the patient received the second cycle of anti-tumor therapy, consisting of albumin-bound paclitaxel (400 mg) and carboplatin (450 mg). To avoid the recurrence of IMH, tislelizumab was not used, and no liver injury or HBVr was observed during subsequent follow-up.

**TABLE 1:** Details of glucocorticoid use in this patient.

Time	Drug	Dose	Frequency	Route of administration
2026.05.27–2026.06.01	Methylprednisolone	30 mg	BID	Intravenous drip
2026.06.02–2026.06.05	Methylprednisolone	20 mg	BID	Intravenous drip
2026.06.06–2026.06.12	Prednisone	40 mg	QD	Peroral
2026.06.13–2026.06.19	Prednisone	30 mg	QD	Peroral
2026.06.20–2026.06.26	Prednisone	20 mg	QD	Peroral
2026.06.27–2026.07.03	Prednisone	10 mg	QD	Peroral

When switching from intravenous methylprednisolone to oral prednisone, methylprednisolone 1 mg is the equivalent dose of prednisone 1.25 mg. Abbreviate: BID, twice a day; QD, once a day.

After thorough information gathering, we summarized the timeline of the appearance, diagnosis, and treatment of HBVr and IMH in this patient (Figure 1). Based on this timeline, we subsequently evaluated the causal relationship between tislelizumab and the occurrences of both HBVr and IMH. Using the Naranjo Adverse Drug Reaction Probability Scale (Naranjo scale) to evaluate the causal relationship between tislelizumab and HBVr (Naranjo et al., 1981), the result showed that the causality is “possible (total score: +3)” (Table 2). Using the Roussel Uclaf Causality Assessment Method (RUCAM) scale to evaluate the causal relationship between tislelizumab and IMH (Danan and Teschke, 2015), the result showed that the causality is “probable (total score: +7)” (Table 3).

**FIGURE 1:**
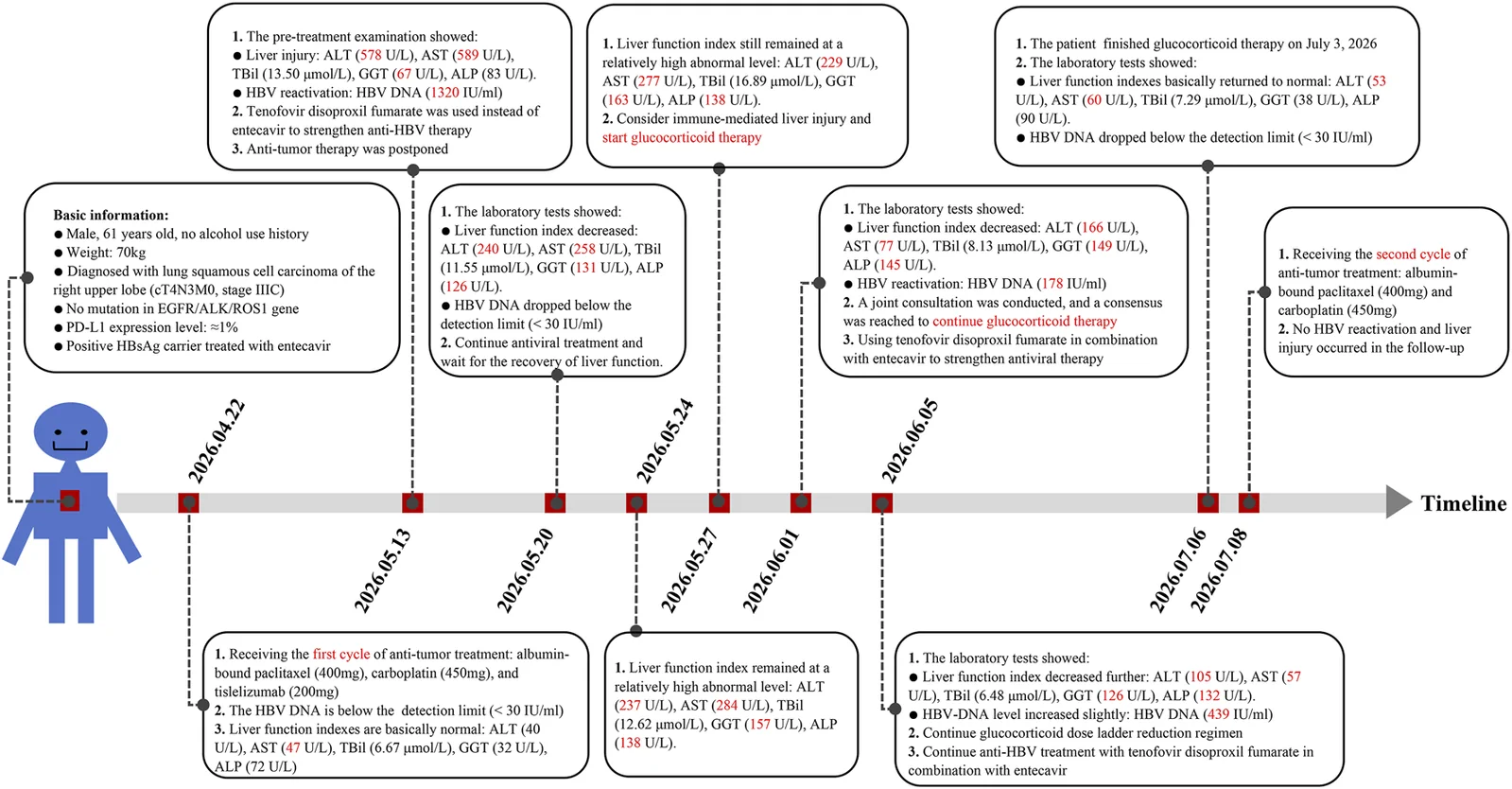
Timeline of diagnosis and treatment of reported patients. Abbreviate: ALT, alanine aminotransferase; AST, aspartate aminotransferase; TBil, total bilirubin; GGT, gamma-glutamyltransferase; ALP, alkaline phosphatase; HBV, hepatitis B virus; HBsAg, hepatitis B surface antigen. Note: Important information is highlighted in red. The normal upper limit value of ALT, AST, TBil, GGT, and ALP are 50 U/L, 40 U/L, 20 μmol/L, 60 U/L, and 125 U/L, respectively.

**TABLE 2:** Details of the causal assessment score of the Naranjo scale.

Question	Scoring criteria			Score and reason
	Y	N	UK	
1. Are there previous conclusive reports on this reaction?	+1	0	0	Score: +1. Reason: A previous study showed that about 5.3% of HBsAg-positive cancer patients developed HBVr after exposure to ICIs.
2. Did the adverse event appear after the suspected drug was administered?	+2	−1	0	Score: +2. Reason: The HBVr was detected 21 days after tislelizumab administration.
3. Did the adverse event improve when the drug was discontinued or a specific antagonist was administered?	+1	0	0	Score: 0. Reason: HBVr a special infection-related adverse event, so it is difficult to evaluate whether HBV inhibition comes from antiviral therapy or tislelizumab discontinuation.
4. Did the adverse event reappear when the drug was readministered?	+2	−1	0	Score: 0. Reason: Tislelizumab was not used again.
5. Are there alternative causes that could on their own have caused the reaction?	−1	+2	0	Score: −1. Reason: Chemotherapy used in conjunction with tislelizumab may also lead to HBVr.
6. Did the reaction reappear when a placebo was given?	−1	+1	0	Score: 0. No placebo was given.
7. Was the drug detected in blood or other fluids in concentrations known to be toxic?	+1	0	0	Score: 0. The relevant tests were not carried out.
8. Was the reaction more severe when the dose was increased or less severe when the dose was decreased?	+1	0	0	Score: 0. The relevant tests were not carried out.
9. Did the patient have a similar reaction to the same or similar drugs in any previous exposure?	+1	0	0	Score: 0. The patient is using ICIs for the first time.
10. Was the adverse event confirmed by any objective evidence?	+1	0	0	Score: +1. The HBV-DNA baseline is below the detection limit before using tislelizumab. After 21 days, the HBV-DNA detection result is 1320 IU/mL, which objectively indicates that HBV was reactivated.
Total score	+3			

Based on the total score of the Naranjo scale, the causality between adverse events and drug use is classified into “definite (total score ≥ 9)”, “probable (total score 5–8)”, “possible (total score 1 to 4)”, and “doubtful (total score ≤ 0)”. Abbreviate: Y, yes; N, no; UK, unknown; HBV, hepatitis B virus; HBVr, hepatitis B virus reactivation; HBsAg, hepatitis B surface antigen; ICIs, immune checkpoint inhibitors.

**TABLE 3:** Details of the causal assessment score of the RUCAM scale.

Evaluation item	Selected option (score)	Reason
1. Time to onset from the beginning of the drug	5–90 days (+2)	Liver injury was found 21 days after tislelizumab administration.
2. Course of ALT after cessation of the drug	Decrease ≥ 50% within 30 days (+2)	Liver injury (ALT 578 U/L) was first detected on 13 May 2026, prompting the postponement and eventual discontinuation of tislelizumab. From May 13 to 5 June 2026, ALT decreased to 105 U/L, representing a reduction of more than 50% over a period of 23 days.
3. Risk factors	Age ≥ 55 years (+1)	This patient is a 61-year-old man without alcohol use.
4. Concomitant drugs	Concomitant drug with incompatible time to onset (0)	The concomitant drugs included albumin-bound paclitaxel, carboplatin, ondansetron, esomeprazole, metoclopramide, fosaprepitant, dexamethasone, nadroparin, and furosemide. Among them, albumin-bound paclitaxel and carboplatin have potential hepatotoxicity, but two main reasons argue against a compatible temporal relationship with the onset of liver injury. First, after completion of a single dose of chemotherapy, the situation can be regarded as a form of drug cessation. In the RUCAM scale, “time to onset from cessation of the drug” is regarded as an alternative evaluation item to “time to onset from the beginning of the drug”. For non-slowly metabolized chemicals, “≤ 15 days” serves as the cut point for defining a compatible temporal relationship with the onset of liver injury. Therefore, the fact that liver injury was first detected 21 days after chemotherapy does not meet the criterion for “time to onset from cessation of the drug.” second, although the actual onset time of liver injury is uncertain, liver injury induced by albumin-bound paclitaxel and carboplatin is typically acute and self-limiting, usually recovering within one to 2 weeks. In this case, liver function remained significantly abnormal 21 days after chemotherapy (ALT: 578 U/L), also making it difficult to reasonably attribute the liver injury at this time point to albumin-bound paclitaxel and carboplatin. Similarly, other concomitant drugs (ondansetron, esomeprazole, metoclopramide, fosaprepitant, dexamethasone, nadroparin, and furosemide) are only used for a short time during chemotherapy, so they are also considered to have an incompatible temporal relationship with the onset of liver injury.
5. Search for alternative causes	6 or 5 causes of group I ruled out (0)	Based on the existing virus serological test results, HCV and HEV are ruled out. Based on medical history, alcoholism and acute recent hypotension history are ruled out. The hepatobiliary sonography is normal, so organic liver lesions are basically ruled out. In general, 5 causes of group I are reasonably ruled out. In addition, the liver injury is persistent after HBV-DNA is undetectable, so HBV is not a highly probable alternative cause.
6. Previous hepatotoxicity of the drug	Reaction labelled in the product characteristics (+2)	Immune-mediated hepatotoxicity is an adverse reaction labelled in the package insert of tislelizumab.
7. Response to unintentional re-exposure	Other situations (0)	There is no re-exposure information available.
Total score	+7	

Based on the total score of RUCAM, scale, the causality between liver injury and drug use are classified into “excluded (total score ≤ 0)”, “unlikely (total score 1–2)”, “possible (total score 3–5)”, “probable (total score 6–8)”, and highly probable (total score ≥ 9). Abbreviate: ALT, alanine aminotransferase; HBV, hepatitis B virus; HCV, hepatitis C virus; HEV, hepatitis E virus.

## 3 Discussion and conclusion

With the wide application of ICIs, the management of irAEs has gradually become an important clinical topic. In anti-tumor clinical practice, both HBVr and IMH are events that may occur during the use of ICIs. In this case report, we described a rare and clinically challenging irAE scenario in which HBVr masked the underlying IMH.

In this patient, the initial presentation of liver injury is considered a manifestation of the combined effects of HBVr and IMH. However, the accompanying detectable HBV-DNA (1320 IU/mL) makes the HBVr the most recognizable cause of liver injury. After switching from entecavir to tenofovir disoproxil fumarate to strengthen antiviral treatment, the undetectable HBV-DNA and rapidly decreasing transaminases further confirmed HBVr as one of the culprits of liver injury. So far, there is no specific test method to identify IMH, and its diagnosis still mainly relies on confirming the compatible temporal sequence between ICI exposure and liver injury, and then reasonably excluding all other possible causes (Peeraphatdit et al., 2020). In the context of HBVr-induced liver injury, the IMH is perfectly masked, making an accurate and timely diagnosis difficult. The confirmation of IMH as the co-culprit lay in the fact that, after an initial rapid decline in transaminase following antiviral therapy, the patient’s transaminase maintained a relatively high abnormal level with little change in the next two liver function tests despite tenofovir disoproxil fumarate continuing. Existing studies have confirmed that a long transaminase normalization course (mean time: 49–60 days) and favorable responsiveness to GCs are important clinical features of IMH (Triantafyllou et al., 2025). In this patient, the persistent abnormal liver function with undetectable HBV-DNA served as a clue suggesting the coexistence of IMH, while the rapid recovery of liver function after GC use confirmed our judgment. However, due to the coexistence of HBVr, the interval between the initial detection of liver injury and the initiation of GC therapy was prolonged to 14 days, which led to a frustrating delay in IMH treatment.

According to the current guidelines, GCs are the mainstay of treatment for IMH (Dougan et al., 2021; Schneider et al., 2021; Haanen et al., 2022). However, GCs are also a well-recognized risk factor for HBVr, as they can not only universally suppress the host immune responses against the virus, but also directly stimulate HBV replication by binding to the GC response element in the HBV genome (Zhou et al., 2022). Generally, the recommended GC administration strategy needs to be determined according to the grade of IMH. According to the CTCAE terminology criteria, the AST of this patient (277 U/L) was higher than 5 times the upper limit of normal when preparing to use GCs, so the severity grade was G3 (Triantafyllou et al., 2025). For IMH patients at grade G3, it is recommended to administer 1–2 mg/kg methylprednisolone. The weight of this patient is 70 kg, so at least 70 mg/d of methylprednisolone should be given at the initial stage of GC treatment. Judging from this dose standard, an intravenous drip of methylprednisolone 30 mg twice a day is not a preferred dose, as a low initial GC dosage regimen may increase the potential risk of IMH treatment failure and should be avoided in clinical practice.

Unexpectedly, the methylprednisolone 60 mg daily improved liver function rapidly despite being given a relatively low dose, but the HBV-DNA levels still switched from undetectable (< 30 IU/mL) to detectable (178 IU/mL) again. According to the AGA 2025 clinical practice guideline on the prevention and treatment of HBVr in at-risk individuals (Ali et al., 2025), the criteria for HBVr are defined as either the *de novo* appearance of HBV-DNA in a patient with previously undetectable HBV-DNA or a 10-fold increase in HBV-DNA value compared with their baseline. According to this standard, the patient was considered to have experienced a second episode of HBVr, despite no transaminase flare. Notably, virologic breakthrough can also lead to a low-level elevation in HBV-DNA, but the following reasons prevented us from including it as a culprit. First, the essence of virologic breakthrough and HBVr are entirely different. Virologic breakthrough is commonly regarded as a potential clinical manifestation of drug resistance, while HBVr typically reflects the loss of HBV immune control in patients receiving immunosuppressive therapy (Terrault et al., 2018; Ali et al., 2025). Tenofovir disoproxil fumarate is an anti-HBV agent with a high barrier to resistance, and a previous 8-year study found no evidence of resistance occurring to HBV and demonstrated that the virologic breakthroughs during treatment were largely attributed to poor medication adherence (Liu et al., 2017). For this patient, medication adherence was confirmed, and the likelihood of drug resistance was also extremely low, so we tend to rule out virologic breakthrough as the primary cause of the elevated HBV-DNA. Furthermore, the known HBVr risk, the reasonable temporal relationship, and no other concomitant immunosuppressive drugs also strongly support that GC is the main trigger for the second episode of HBVr.

In this situation, HBVr posed another therapeutic dilemma to IMH, namely, that IMH needs to continue to use GC, but it may also result in acute exacerbation of HBV infection. So far, only few published case reports have documented the co-occurrence of IMH and HBVr during ICI therapy. Baumert LS et al. reported a lung cancer patient who developed IMH and HBVr after nivolumab treatment, characterized by jaundice, markedly elevated peak ALT/AST levels (4613/3797 U/L), panlobular hepatitis, and a high HBV viral load (9.21 × 108 IU/mL) (Baumert et al., 2024). Despite the severity of liver injury, the patient achieved a smooth recovery after treatment with entecavir and GC. By contrast, the patient in our case experienced a second episode of HBVr during GC and antiviral treatment, which made the case unique. At present, guidelines on IMH management do not give clear treatment suggestions for this special situation (Dougan et al., 2021; Schneider et al., 2021; Haanen et al., 2022). The successful resolution of this dilemma relied on a multidisciplinary joint consultation involving gastroenterologists, oncologists, and clinical pharmacists. After the discussion, we reached a consensus that continuing GC therapy is beneficial to restore liver function and restart anti-tumor therapy, while the risk of HBV activation is relatively controllable under the setting of gradually decreasing GC and dual-drug antiviral therapy. Actually, dual-drug therapy (entecavir plus tenofovir) is not a preferred antiviral regimen recommended by the guidelines, but the following two reasons prompt us to make and implement this strategy. First, in the setting of occurring antiviral resistance, dual-drug therapy is an optional strategy to enhance antiviral therapy recommended by the guidelines (Terrault et al., 2018; Cornberg et al., 2025). Second, the treatment of IMH requires ensuring that ongoing GC therapy is not interrupted by further HBV activation. However, due to the disturbance of anti-tumor drugs and GC on immunity, previous monotherapy with either entecavir or tenofovir has been demonstrated to be inadequate for complete suppression of HBV replication. Therefore, although there is no definitive evidence of drug resistance, we still chose to combine tenofovir with entecavir to enhance antiviral effect, thereby mitigating the risk of HBV acute exacerbation associated with ongoing GC therapy.

Another noteworthy detail is that although liver function continued to improve, HBV-DNA showed a transient increase (from 178 to 439 IU/mL) 4 days after the initiation of dual antiviral therapy. The mechanism underlying this abnormal clinical phenomenon of dissociation remains unclear. We speculated that two factors may be involved. First, continued GC therapy further alleviated IMH, improving liver function. However, given that GC had already induced HBRr, its continued use also further stimulated HBV replication. Second, as a newly added agent, entecavir had not yet reached its full antiviral potency within the first 4 days, as it requires 6–10 days to achieve steady-state plasma concentration (Scott and Keating, 2009). Therefore, HBV-DNA may exhibit a controllable transient increase during the initial phase of dual antiviral therapy, which is also the reason why we did not implement additional clinical intervention. Subsequently, the successful recovery of the patient’s liver function confirms the appropriateness of the overall treatment strategy and also highlights the importance of a multidisciplinary approach.

After the patient finished the second cycle of anti-tumor treatment, we retrospectively assessed the causal relationship between tislelizumab use and the development of HBVr and liver injury, respectively. The evaluation results from the Naranjo scale showed that the occurrence of HBVr followed a temporal sequence after the use of tislelizumab, but concomitant chemotherapy played a similar role in the HBVr (Naranjo et al., 1981). Therefore, in the absence of re-exposure information, it is difficult for us to determine whether HBVr is caused by tislelizumab, chemotherapy, or a joint action. By contrast, the evaluation result from the RUCAM scale showed a much stronger causal relationship between tislelizumab and liver injury. Although the re-exposure information is also unavailable, the known hepatotoxicity, reasonable onset and recovery time of liver injury, favorable responsiveness to GCs, and most importantly, reasonable exclusion of most possible causes became solid evidence for establishing a causal relationship between tislelizumab and liver injury (Danan and Teschke, 2015; Triantafyllou et al., 2025).

Several clinical lessons can be drawn from our case. First, patients receiving ICI treatment may suffer HBVr and IMH synchronously, and HBVr can mask the underlying IMH, which may lead to the delay of diagnosis and treatment of IMH. Second, GC therapy for IMH may also induce HBVr again, and a multidisciplinary approach is important to develop reasonable treatment strategies in such a therapeutic dilemma. Third, for patients occurring HBVr during GCs therapy for IMH, it may be a potentially beneficial option to continue GCs treatment on the premise of strengthening anti-HBV treatment. However, the limitations of this case report have to be mentioned. First, based on limited information, the causal relationship between tislelizumab and the first episode of HBVr cannot be fully established. Second, re-exposure information is one of the strongest types of evidence in the causality assessment (Hammad et al., 2023), but this information is unavailable due to the patient stopping tislelizumab treatment for safety concerns. Finally, as a single case report, it is unknown whether patients with similar conditions can benefit from the treatment strategies employed in this case. When dealing with such a special patient, clinical decisions still need to be made after multidisciplinary discussions.

In conclusion, this case highlights that IMH and HBVr can occur concurrently in patients receiving ICIs, even in the presence of antiviral prophylaxis. The coexistence of HBVr and IMH can greatly complicate both diagnosis and treatment of IMH. In this clinical scenario, the multidisciplinary approach, close monitoring of liver function and HBV-DNA, and a carefully balanced treatment strategy combining GCs with intensified antiviral therapy are essential to realize the optimal management for IMH combined with HBVr.

## Data Availability

The original contributions presented in the study are included in the article/supplementary material, further inquiries can be directed to the corresponding authors.
